# Current Status of Dapagliflozin in Congestive Heart Failure

**DOI:** 10.7759/cureus.29413

**Published:** 2022-09-21

**Authors:** Gopal Palandurkar, Sunil Kumar

**Affiliations:** 1 Department of Medicine, Jawaharlal Nehru Medical College, Datta Meghe Institute of Medical Sciences, Wardha, IND

**Keywords:** type 2 diabetes, sglt2 inhibitor, dapagliflozin, epidemiology of hf, congestive heart failure

## Abstract

Heart failure is a prominent clinical condition and a top concern as a widespread health issue. The incidence of heart failure is rising alarmingly on a global scale. Heart failure significantly strains the whole healthcare system financially, degrading the patient's quality of life and increasing the risk of morbidity and mortality. Heart failure treatment has changed over time with ongoing research and the development of new medications and equipment.

Recently, the FDA and European Union (EU) approved the drug dapagliflozin, which is an inhibitor of sodium-glucose cotransporter 2 (SGLT-2i), for treating people with cardiovascular conditions and symptomatic heart failure (HF). In this review article, we will find out whether Dapagliflozin, when given at a dose of 10 mg/day in people with type 2 diabetes and in those without type 2 diabetes who have or are at risk for atherosclerotic alterations, can considerably lower the risk of cardiovascular mortality or hospitalization for HF. In the presence of concomitant HF therapies, dapagliflozin's benefits remained. Dapagliflozin's overall safety profile was comparable to its safety profile for other applications. It was often well tolerated in patients in a study group. In this review article, dapagliflozin is found to be the well-tolerated and effective novel treatment of choice for symptomatic HF. Because of the scarcity of research on dapagliflozin, it was necessary to provide data to help reduce the mortality of patients while providing further guidance on the clinical medication of dapagliflozin.

## Introduction and background

Heart failure (HF) is the most common substantial financial and medical burden in the world [1,2]. Since mortality is still high and life quality is poor, it is a focus of continuous research [3,4]. We update recently released data and findings in this article. HF has emerged as a severe global health problem with an incidence rate of more than 37.7 million individuals worldwide [5]. The burden is quickly growing, and it is predicted that by 2030, there will be a 25% increase in HF patients [6]. In well-sustained nations, the prevalence of HF in adults varies from 1.1% to 2.3%. As individuals age [7,8], it steadily rises until it reaches 10% or higher in those over 80; age and type 2 diabetes mellitus (T2DM) are known to increase the incidence of HF [9]. There is a well-established link between HF and T2DM. No medication available now lowers the incidence of HF in those with T2DM. Recently, decreasing cardiovascular risk in T2DM has been achieved mainly by inhibiting sodium-glucose cotransporter 2. A new type of hypoglycaemic agent named dapagliflozin belongs to a class of sodium-glucose co-transporter 2 inhibitors (SGLT-2i) [10]. Other medications in the SGLT-2i group, primarily in patients diagnosed with type 2 diabetes and those with well-established cardiovascular disease, have demonstrated lower cardiovascular effects, including a lower likelihood of hospitalization for heart failure.

Dapagliflozin can lower blood sugar levels without being affected by peripheral tissue insulin resistance or insulin secretion. SGLT-2i inhibits the reabsorption of glucose by the renal tubules. Therefore, the excretion of glucose is increased in urine and lowers blood sugar levels by considerably reducing the functional activity of the proximal renal tubules. SGLT-2i drugs also have other effects than hypoglycaemic effects. For example, empagliflozin and canagliflozin have been proven to have additional cardiovascular protection. They can reduce the occurrence of cardiovascular events [7,11]. Dapagliflozin, a related medicine, has also been shown in specific trials to help with cardiovascular disease. This impact is independent of hypoglycemia. Therefore, it has become an essential therapy option for decreasing cardiovascular risk. In addition, people worry about the current status of Dapagliflozin and its potential benefit in reducing cardiovascular risks. As a result, prolonging the life duration of HF patients, reducing mortality, and improving the outcome are critical challenges that need to be addressed in the current era. This paper offers a systematic assessment of a program to examine the safety and efficacy of dapagliflozin in heart failure, as well as to give a suitable foundation for subsequent clinical pharmacological recommendations to prevent needless traps.

Method

**Figure 1:**
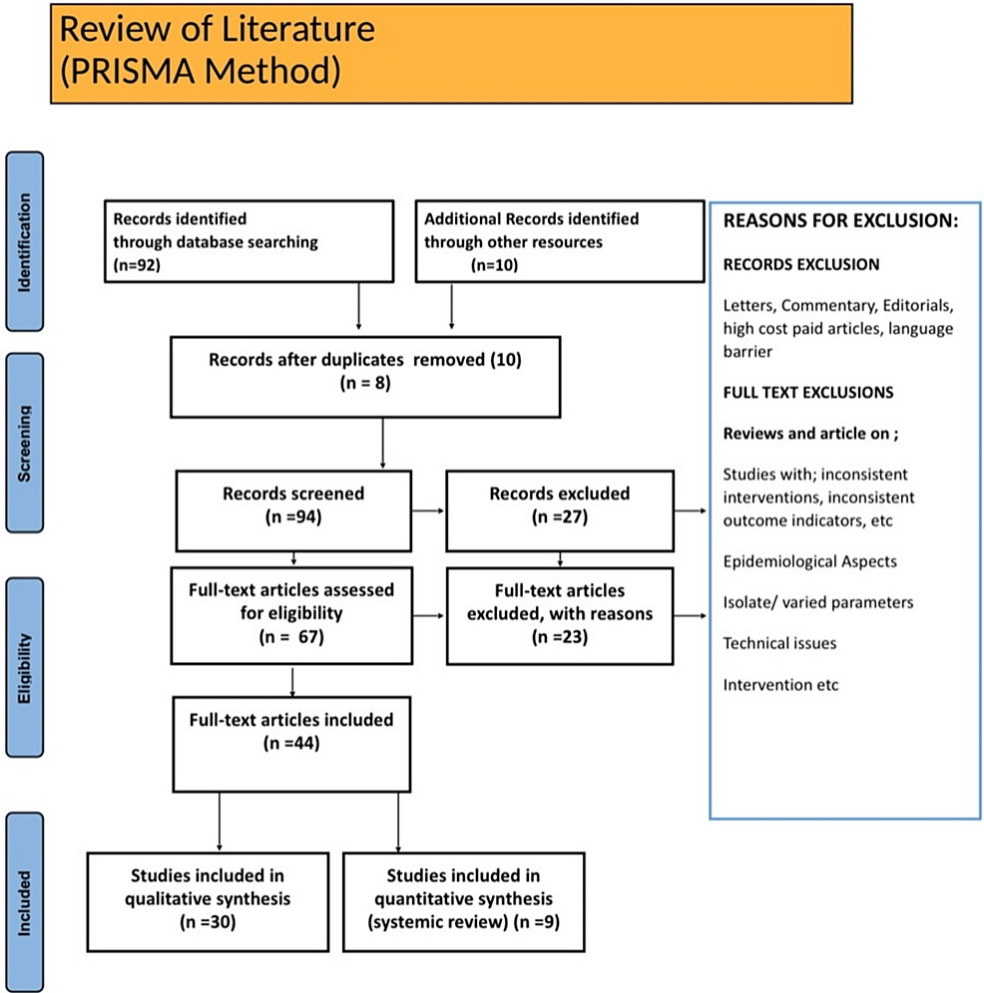
Flow diagram of literature review

The following goals are being pursued with this review: Current status of SGLT2i dapagliflozin in congestive heart failure. A search of the English-language literature was done using the electronic databases PubMed, Embase, Google Scholar, and Medline. And the search terms were Congestive heart failure or Epidemiology of HF or Dapagliflozin or SGLT-2i or type 2 diabetes. The writers' personal knowledge and experience in the field supported the archiving of relevant papers. Articles that match the following criteria are included in this review: studies in English; studies from the last ten years; and studies devoted entirely to congestive HF, dapagliflozin, and SGLT-2i. Research methodology by PRISMA (Preferred Reporting Items for Systematic Reviews and Meta-Analyses) method is shown below in Figure 1.

## Review

HF and classification

**Figure 2:**
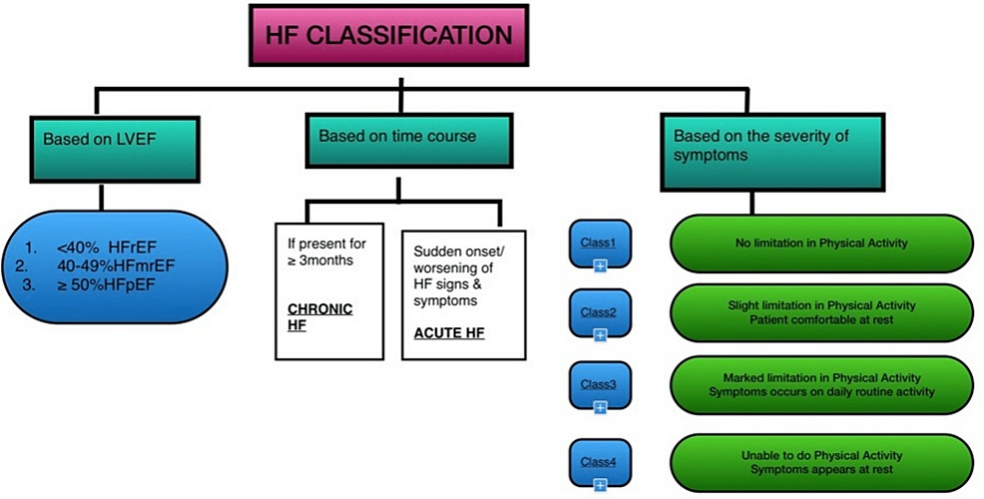
Classification of HF HF=heart failure; LVEF=left ventricular ejection fraction; HfrEF=heart failure with reduced ejection fraction; HfmrEF=heart failure with mid-range ejection fraction; HFpEF=heart failure with preserved ejection fraction

HF is a complex clinical syndrome that underlines the inability of the heart to perform its circulatory function with the desired efficiency due to structural and/or functional (systolic or diastolic) alterations [12,13]. Not even a single, widely accepted HF categorization has been followed to date. Figure 2 Summarises the commonly followed classification systems in HF management [12-14].

Other terms commonly used in HF are as follows [12,13]: Stable HF - After at least a month of therapy, a patient is considered to have stable HF if their symptoms and indications have not altered considerably. Decompensated HF - Decompensated HF is when a chronic previously stable patient's condition worsens abruptly or gradually. New-onset HF - A person with newly developed/de novo heart failure may have subacute or acute (gradual) symptoms. Advanced HF - Patients with recurring decompensation, significant cardiac dysfunction, and severe symptoms while receiving excellent conventional medical care fall under this category.

Epidemiology

A complex syndrome of multi-morbidity and geriatric symptoms is also linked to age-related increases in the incidence and prevalence of HF [15,16]. In terms of gender distribution, women are twice as likely as men to develop HFpEF (heart failure with preserved ejection fraction) [17]. Globally, between 17 to 45 percent of hospitalized heart failure patients die within a year of being diagnosed, with the majority dying within five years [18]. Around two to seventeen percent of those brought to a hospital with heart failure die while there [19-21]. Survival rates are higher in outpatient clinics, where patients often have fewer severe symptoms than in hospitals [20,22,23]. Even the most advanced medicines may improve symptoms in many people without reducing disease progression or extending life [23,24].

Compared to North America and Western Europe, Asian patients are much younger. With the exception of Japan, several Asian nations have a higher adjusted risk of cardiovascular (CV) mortality. Also, Jones et al. published lasting results for 1.5 million steady chronic heart failure patients, consisting of 60 non-interventional studies. Even if five-year survival rates have increased from 29.1% to 59.7% between 1970-1979 and 2000-2009, the number of deaths is still too high and is greater than for other types of cancer [25-27]. Making a risk prediction model is still tricky since global results might underestimate or overestimate mortality in certain countries [28].

Pharmacological properties

Dapagliflozin (SGLT-2i) has been approved for the treatment of patients with symptomatic HF in a number of countries, including the United States [29] and the European Union [30]. Dapagliflozin's pharmacological characteristics have already been evaluated in-depth [31-33] and are also presented in Table 1. Table 2 shows the contraindications, warnings, precautions, and adverse effects of the drug dapagliflozin [28].

**Table 1:** Overview of dapagliflozin's principal pharmacologic characteristics A consideration of these indications is outside the purview of this study, however, dapagliflozin is also authorised for the treatment of type 2 diabetes (T2DM) [31,32], type 1 diabetes (T1DM) [33], and chronic kidney disease (CKD). SGLT=Sodium-glucose cotransporter; DAP=dapagliflozin; *UGT1A9=UDP Glucuronosyltransferase Family 1 Member A9*;* *AUC=area under curve

Overview of dapagliflozin's principal pharmacologic characteristics [13,14]
Pharmacodynamic Properties
A very powerful (Ki 0.55 nM) and irreversible substance that is > 1400 times more selective for SGLT2 than SGLT1 inhibits SGLT2 (the prime intestinal glucose transporter)
decreasing renal glucose reabsorption and increasing urine glucose excretion to lower plasma glucose levels
increases sodium transport to the distal tubule while reducing salt absorption, which is expected to enhance tubuloglomerular feedback and decrease intraglomerular pressure
SGLT2-induced natriuresis and diuresis cause reduced interstitial fluid volume, blood volume, afterload, and preload on the heart. The volume and pressure at the conclusion of the left ventricular diastole are likewise reduced
reduces bodyweight and increases hematocrit
When supratherapeutic doses of up to 500mg were used there was no clinically meaningful effect seen over QTc interval
Pharmacokinetic properties
0.1-500 mg dosing range; dose-proportional exposure; no pharmacokinetic change after daily treatment for up to 24 weeks
After a single 10 mg dose, there is a 78 percent absolute oral bioavailability, and it is quickly absorbed after oral administration; Cmax is attained in 2 hours (fasted state); percentage of drug that is protein bound is 91. On average the volume of distribution is 118L.
A minor clearance pathway is Cytochrome P450-mediated metabolism; extensively metabolised by *UGT1A9* converts its primary inactive metabolite in the liver and kidney (dapagliflozin 3-O-glucuronide)
Mostly excreted through the urinary system; after a single 10 mg dosage, 12.9 hours is the average plasma terminal half-life. About 21% of the radiolabelled dosage recovered in faeces and the amount radiolabelled dose that is recovered in urine is 15%. in total about 75 % of the radiolabelled dose is recovered
Special populations: No race, age, or body weight-related variations in DAP pharmacokinetics, and the mean steady-state AUC is anticipated about approximately 22% times higher in women except in men. The mean Cmax and AUC are up to 40 and 67 percent higher in individuals with severe hepatic impairment compared to healthy matched controls, respectively. Mean systemic exposures to DAP are higher in individuals with mild, moderate, or severe abnormal kidney function compared to those with normal renal function.
Drug interaction: Doesn't appreciably inhibit organic anion transporters-3, organic anion transporters-2, organic anion transporters, or organic cation transporters-1; nor does it stimulate Cytochrome P450 1A2, Cytochrome P450 2B6, or Cytochrome P450 3A4; neither does it inhibit Cytochrome P450 1A6, Cytochrome P450 2A2, Cytochrome P450 2C8, Cytochrome P450 2 B6, Cytochrome P450 2C19, Cytochrome P450 2C9, or Cytochrome P450 2D6. DAP had no impact over the pharmacokinetics of bumetanide, pioglitazone, simvastatin, metformin, sitagliptin, or valsartan's, hydrochlorothiazide, glimepiride. Warfarin or digoxin's pharmacokinetics were unaffected by DAP (including its anticoagulant effects) Mefenamic acid (an inhibitor of *UGT1A9*) increased DAP exposure by 55% whereas rifampicin (a medicine) lowered DAP exposure by 22%; nevertheless, neither medication had a clinically significant impact on 24-hour urine glucose levels. DAP may enhance the risk of hypotension and dehydration by amplifying the diuretic effects of loop diuretics and thiazides

**Table 2:** Contraindications, warnings, precautions and adverse effects of drug dapagliflozin SGLT2=sodium-glucose cotransporter 2

Dapagliflozin contraindications, warning, precautions, and adverse effects [28]
Contraindications
History of a serious hypersensitivity reaction such as anaphylactic reactions or angioedema; patient on dialysis
*Precautions* *and Warnings*
Dapagliflozin-induced intravascular volume depletion may manifest as clinical hypotension or rapid, transient changes in creatinine. Before initiating dapagliflozin, individuals should be assessed for renal function and volume status. Keep a watch out for hypotension symptoms and renal function after commencing treatment
In placebo-controlled investigations of individuals with type 1 diabetes, those who took SGLT2 inhibitors had a greater chance of developing ketoacidosis than those who got a placebo. Ketoacidosis is a dangerous illness that can be fatal and requires emergency hospitalisation. Patients on dapagliflozin have died as a result of ketoacidosis. Dapagliflozin should not be used to treat patients with type 1 diabetes. If patients on dapagliflozin display symptoms and signs of severe metabolic acidosis, they should be evaluated for ketoacidosis
Patients using dapagliflozin have developed significant urinary tract infections, including pyelonephritis and urosepsis, requiring hospitalisation. Urinary tract infections are more prevalent when using SGLT2 inhibitors. Examine patients for signs of urinary tract infections and manage appropriately
Dapagliflozin usage increases the likelihood of genital mycotic infections. Patients who had a history of genital mycotic infections were more likely to have them
Fournier’s Gangrene (Necrotising fasciitis of the perineum), a rare but serious necrotising infection that needs rapid surgical intervention, has been documented in patients with diabetes mellitus using SGLT2 inhibitors. Serious effects include hospitalisation, several operative procedures, and even death have been reported. Patients must be managed appropriately.
It is well known that insulin and insulin secretagogues can lead to hypoglycemia. If dapagliflozin is used with insulin or an insulin secretagogue, the risk of hypoglycemia may worsen. Therefore, when these medications are combined with dapagliflozin, a lower dosage of insulin or an insulin secretagogue may be necessary to reduce the risk of hypoglycemia
Adverse Effects
Volume depletion, diabetic ketoacidosis, pyelonephritis and urosepsis, hypoglycemia caused by insulin, and insulin secretagogues, genital mycotic infections, and necrotising fasciitis of the perineum are all possible adverse effects

Clinical effectiveness of dapagliflozin

A major topic discussed in this section is the current efficacy of dapagliflozin as a treatment goal in treating patients suffering from HF. In this section, these statistics are briefly addressed.

a) DAPA-HF (Dapagliflozin and Prevention of Adverse Outcomes in Heart Failure) Trial

Kansas City Cardiomyopathy Questionnaire (KCCQ) total symptom scores (TSS) are used to divide patient populations patient population, and Cox proportional hazards models were applied to analyze how Dapagliflozin affected various clinical outcomes in each subgroup. Dapagliflozin's effects were examined on the clinical summary score and KCCQ-TSS on the overall summary score. Based on an 18-month recruitment period and a 24-month average follow-up period, it was estimated that 4,500 patients would produce the required number of primary events, with an annual event rate of 11% in the placebo treatment group [34]. Given the 30% relative risk reduction in HF hospitalisation seen in other studies, a treatment effect size of 20% was chosen as being both clinically significant and relatively conservative. To evaluate the percentage of dapagliflozin-treated patients against the patients treated with placebo, there were clinically significant improvements in KCCQ at eight months, and responder analyses were done at four and eight months [34]. KCCQ was accessible for 4,443 people at baseline median KCCQ-TSS, which was 77.1. In all tertiles of the KCCQ-TSS, dapagliflozin had equivalent effects to a placebo in the decline in cardiovascular mortality and the deterioration of heart failure. At eight months, patients receiving dapagliflozin had improved more than those receiving placebo in terms of mean KCCQ-TSS, clinical summary score, and overall summary score (2.81, 2.53, and 2.31 points greater than placebo; P0.0001 for all) [34]. Some of the patients receiving dapagliflozin were downgraded in their KCCQ-TSS. Many participants experienced at least smaller, medium, and more considerable advancements; 14, 15, and 18 are the necessary numbers to treat. Treatment with dapagliflozin decreased mortality and hospitalizations for HF ranging from the DAPA-HF study's baseline KCCQ levels. Additionally, it reduced the severity of symptoms, boosted functional status, and improved the quality of life in HFrEF (heart failure with reduced ejection fraction) patients [34].

b) DECLARE-TIMI (Dapagliflozin Effect on Cardiovascular Events-Thrombolysis in Myocardial Infarction) 58 Trial

All patients were asked about their baseline heart failure status for the DECLARE-TIMI 58 study. Whenever available, EF was also measured. EF 45% was used to define HFrEF. Out of 17,160 patients, 671 constrained (HFrEF) heart failure with such a reduced EF, 1,316 constrained heart failure without such a documented decreased EF, and 15,173 (88.4%) had never before had heart failure. Dapagliflozin's therapeutic efficacy was similar in individuals with HF who had no known decreased EF as well as those without HF. Patients with HFrEF had against those without HFrEF reduced chance of cardiovascular mortality and HF. Dapagliflozin reduced cardiovascular death exclusively in participants constrained with HFrEF; still, no definitive effect is seen in participants without HFrEF. In contrast, it decreased the chances of HF in those participants with HFrEF and those without HFrEF. We discovered that dapagliflozin significantly reduced the incidence of heart failure in HFrEF-positive and -negative patients, cardiovascular death, and all other causes related to mortality in HFrEF-positive patients [35].

c) DEFINE-HF (Dapagliflozin Effects on Biomarkers, Symptoms and Functional Status in Patients with HF with Reduced Ejection Fraction) Trial

The investigator-initiated, multi-centre, randomized controlled trial known as DEFINE-HF included: a) HF patients with a left ventricular ejection fraction of less than 40%. b) An estimated glomerular filtration rate of less than 30 mL/min/1.73 m2. c) A New York Heart Association (NYHA) class of II to III. d) Elevated natriuretic peptides. Dapagliflozin 10 mg was given to 263 volunteers randomly or a placebo for a period of around 12 weeks. Two primary outcomes were considered: the percent of participants whose overall summary score somewhat on the KCCQ has improved by at least five points due to their HF condition and the mean NT-proBNP (N-terminal pro-b-type natriuretic peptide), or an NT-proBNP drop of at least twenty percent. Patients had persistent, chronic HF, a low ejection fraction, and significant usage of the best available medical care. Average adjusted NT-proBNP levels at six and twelve weeks were similar between dapagliflozin and placebo [36]. The overall summary score on the NT-proBNP or KCCQ has significantly improved; the second dual-primary goal was attained at about 50.4 per cent for placebo-treated patients and over 61.5 percent in dapagliflozin-treated participants. This was because more patients had 12-week NT-proBNP levels that had decreased by 20% and saw a five-point increase in their overall summary score on the KCCQ. The addition of dapagliflozin for 12 weeks did not alter the mean NT-proBNP among HFrEF patients with and without T2DM receiving optimal guideline-directed medical therapy, but it did significantly raise the proportion of patients who had improvements in natriuretic peptides and their disease-specific health status [36].

*d)* *DICTATE-AHF (Efficacy and Safety of Dapagliflozin in Acute Heart Failure) Trial*

The goal is to evaluate the safety and effectiveness of initiating dapagliflozin on individuals with AHF during the first 24 hours of hospitalization versus standard therapy. A total of 240 patients are expected to participate in the prospective, multicenter, open-label, randomized DICTATE-AHF study by the United States. Hypervolemic acute heart failure (AHF) hospitalization, T2DM, and a glomerular filtration rate estimated to be about 31 mL/min/1.73m2 are all requirements for involvement. Dapagliflozin 10 mg per day or organised standard of care is randomly allocated to each patient until the end of day five or till hospital discharge. Both groups under treatment get prescribed doses of insulin and diuretics [37]. The primary outcome is the diuretic response, measured as the weight change divided by the total amount of loop diuretics in 40 mg IV furosemide analogous throughout time. AHF is worsening while an inpatient, hospital readmission of over 30 days for diabetic reasons or changes in NT-proBNP, AHF, and indicators of natriuresis are a few secondary and exploratory outcomes. Safety objectives include the frequency of ketoacidosis, hypo- or hyperglycemia, deteriorating renal function, hypovolemic hypotension, and inpatient mortality [37].

e)* DELIVER (Dapagliflozin Evaluation to Improve the Lives of Patients with Preserved Ejection Fraction Heart Failure) Trial*

DELIVER is a global, parallel-group, multicenter, event-driven, double-blind trial randomized in participants with left ventricular ejection fraction > 40% and chronic heart failure when dapagliflozin 10 mg single dose daily was compared with placebo as well as standard of treatment. Patients with diabetes, without diabetes, heart failure symptoms and signs, a left ventricular ejection fraction (LVEF) of at least 40%, elevated natriuretic peptide levels, and indicators of structural heart disease are eligible [37]. The main goal will be the duration until the first heart failure episode or cardiovascular mortality, which will be analyzed in two independent analyses, encompassing the overall population and those with LVEF of less than 60%. The study will focus on 1117 key events and is event-driven. 6,263 patients in all have been randomly assigned. The SGLT-2i dapagliflozin will be introduced to standard treatment in DELIVER patients with heart failure with preserved or modestly diminished ejection fraction to assess its effectiveness and safety [37].

f) Trial Conducted by Wiviott et al.

Patients with T2DM who had or were at risk for atherosclerotic cardiovascular disease were randomly allocated to either dapagliflozin or a placebo. Major adverse cardiovascular events (MACE), which include ischemic stroke, cardiovascular mortality, and myocardial infarction, were the critical safety outcome. The critical effectiveness outcomes were MACE and a composite of cardiovascular mortality or hospitalization for HF. The secondary efficacy outcomes were the renal composite and death from any cause [10].

The study included 17,160 people, 10,186 of whom were followed for an average of 4.2 years and showed no signs of atherosclerotic cardiovascular disease. In the analysis of primary safety outcomes, dapagliflozin showed the pre-specified criteria in non-inferiority to placebo in MACE. In the two major effectiveness studies, dapagliflozin did not reduce the risk of MACE. However, it reduced the hospitalization rate for heart failure or cardiovascular mortality. A renal event occurred in 6.22 percent and 6.61 percent of the dapagliflozin groups. In comparison, it happened in 5.6 percent of the placebo group. The proportion of genital infections that resulted in drug discontinuation or were considered serious adverse events was more significant with dapagliflozin than with placebo, and diabetic ketoacidosis was more common [10]. In patients with type 2 diabetes who had or were at risk of atherosclerotic cardiovascular disease, dapagliflozin did not produce a higher or lower rate of MACE than placebo; however, it did produce a lower rate of cardiovascular death or hospitalization for HF, a finding that reflects a lower rate of HF hospitalization [10].

e) Trial Conducted by McMurray et al.

Randomly assigning 4,744 patients in this phase three placebo-controlled experiment was done by McMurray et al. with New York Heart Association class 2, 3, 4 heart failure and with an ejection fraction of 40.1% or less to receive dapagliflozin at a dose of 10 mg once per day or placebo, as well as standard of treatment. Two primary outcomes were worsening heart failure or cardiovascular mortality. The main objective met in the placebo group was 502 of 2,371 patients (21.2%). Over a median of 18.2 months in the dapagliflozin group, it was 386 of 2,373 patients (16.3%). Two hundred and thirty-seven patients (10.1%) experienced worsening heart failure in the dapagliflozin group, whereas 326 patients (13.7%) experienced deteriorating heart failure in the placebo group. In the dapagliflozin group, 227 individuals (9.6%) died from cardiovascular disease, and in the placebo group it was 273 patients (11.5%); 276 patients (11.6%), and 329 patients (13.9%) suffered all other causes of death. Regardless of diabetes status, patients with low ejection fraction who received dapagliflozin had a reduced risk of worsening heart failure or dying from cardiovascular causes than those who received a placebo [39].

g) *DAPA-CKD (Dapagliflozin and Prevention of Adverse Outcomes in Chronic Kidney Disease) Trial*

The effects of dapagliflozin on kidney, cardiovascular, and mortality outcomes were compared to the presence of T2DM and any chronic kidney disease (CKD) causes. The DAPA-CKD study participants were randomised to receive dapagliflozin 10 mg once a day or a similar placebo in addition to standard treatment. The estimated glomerular filtration rate (eGFR) of the participants was 25-75 mL/min per 1-73 m2 and the urine albumin to creatinine ratio was 200-5000 mg/g [38]. The primary and secondary outcomes of the DAPA-CKD trial were examined in this study based on the presence or absence of T2DM and the aetiology of CKD. Dapagliflozin reduces the risk of major adverse renal, cardiovascular, and all-cause mortality events in patients with diabetes and non-diabetic CKD. In DAPA-CKD, 11% of CKD patients with and without T2DM had a history of HF [38]. This trial indicated that dapagliflozin was equally beneficial in decreasing the risk of kidney failure, the composite endpoint of hospitalisation for HF or cardiovascular mortality, and death from any cause, independent of baseline HF history. Regardless of a patient's diabetes history, these findings highlight the crucial role that SGLT2 inhibitors play in the primary and secondary prevention of HF in people with CKD. Dapagliflozin was found to be effective in reducing both first-time and recurring hospitalizations. When recurrent occurrences were examined, dapagliflozin particularly reduced the overall number of hospitalizations for HF by 60%. Dapagliflozin decreased mortality in those with and without a history of heart failure in the same way [38].

## Conclusions

This research combines the latest information on HF, including global epidemiology, HF types, and the status of the approved drug dapagliflozin. Despite tremendous advances, cardiovascular disease (HF) remains the primary cause of poor quality of life in persons with cardiovascular illnesses or comorbidities. Since the development of innovative therapy techniques, medications in the SGLT2-i class, such as dapagliflozin, when administered at a dose of 10 mg once daily in symptomatic heart failure have decreased mortality, improved quality of life, and increased life expectancy. This review includes an overview of some of the studies that have been conducted till date, as well as a discussion of their findings. There have been certain studies which show positive reviews in terms of the use of dapagliflozin and still some experiments are currently in progress. Dapagliflozin is frequently well-tolerated and is an effective novel medication in symptomatic HF that has been added to the list of options.
